# Supramammillary projections to the lateral preoptic area drive dopamine release and active behavior

**DOI:** 10.64898/2026.09.01.748391

**Published:** 2026-09-07

**Authors:** Yosuke Arima, Zengyou Ye, Coleman B. Calva, Xia Min, John Gibbons, Jorge Mendoza, Beyonce Getachew, Sarah T. Johnson, Satoshi Ikemoto

## Abstract

The supramammillary nucleus (SuM) is known to promote active, approach-oriented behaviors by engaging ventral tegmental area (VTA) dopamine neurons projecting to the nucleus accumbens (NAc), primarily via a glutamatergic SuM→medial septum (MS) pathway. However, the role of other SuM projections in this process remains unclear. Here we combined anatomical tracing, fiber photometry, chemogenetic, and optogenetic approaches to investigate the SuM→lateral preoptic area (LPO) pathway. We found that SuM→LPO neurons possess extensive collateral projections and are inhibited during consummatory behavior but activated by aversive and unexpected stimuli. Chemogenetic activation of these neurons reduced water intake and immobility during stress, while inhibition enhanced the appetitive phase for ingestive behavior and fear-related immobility. Optogenetic stimulation of SuM→LPO terminals reinforced behavior and elicited dopamine release in the NAc. These findings identify the SuM→LPO pathway as a distinct circuit that, alongside the SuM→MS pathway, engages mesolimbic dopamine signaling to facilitate active behavioral responses to environmental challenges.

**SIGNIFICANCE STATEMENT:**

Active coping with threat or opportunity may depend on the supramammillary nucleus (SuM) to mobilize dopamine-driven approach behavior. This study identifies the lateral preoptic area as a second SuM target capable of driving dopamine release and reinforcing approach behavior, with activity that tracks whether an animal is facing a threat or consuming a reward. Because SuM neurons broadcast to this and other targets through extensive collateral branches, these results reshape how the SuM’s role in active, approach-oriented behavior should be understood — not as a single circuit, but as a distributed one.

## Full Text Availability

The license terms selected by the author(s) for this preprint version do not permit archiving in PMC. The full text is available from the preprint server.

